# *In situ* Proteomic Profiling of Curcumin Targets in HCT116 Colon Cancer Cell Line

**DOI:** 10.1038/srep22146

**Published:** 2016-02-26

**Authors:** Jigang Wang, Jianbin Zhang, Chong-Jing Zhang, Yin Kwan Wong, Teck Kwang Lim, Zi-Chun Hua, Bin Liu, Steven R. Tannenbaum, Han-Ming Shen, Qingsong Lin

**Affiliations:** 1 Interdisciplinary Research Group in Infectious Diseases, Singapore-MIT Alliance for Research & Technology (SMART), 138602, Singapore; 2 Department of Biological Sciences, National University of Singapore, 117543, Singapore; 3 The State Key Laboratory of Pharmaceutical Biotechnology, College of Life Sciences, Nanjing University, Nanjing, 210023, China; 4 Department of Physiology, Yong Loo Lin School of Medicine, National University of Singapore, 117597, Singapore; 5 Department of Chemical and Biomolecular Engineering, National University of Singapore, 117585, Singapore; 6 Departments of Biological Engineering & Chemistry, Massachusetts Institute of Technology, 02139, United States of America

## Abstract

To date, the exact targets and mechanism of action of curcumin, a natural product with anti-inflammatory and anti-cancer properties, remain elusive. Here we synthesized a cell permeable curcumin probe (**Cur-P**) with an alkyne moiety, which can be tagged with biotin for affinity enrichment, or with a fluorescent dye for visualization of the direct-binding protein targets of curcumin *in situ*. iTRAQ^TM^ quantitative proteomics approach was applied to distinguish the specific binding targets from the non-specific ones. In total, 197 proteins were confidently identified as curcumin binding targets from HCT116 colon cancer cell line. Gene Ontology analysis showed that the targets are broadly distributed and enriched in the nucleus, mitochondria and plasma membrane, and they are involved in various biological functions including metabolic process, regulation, response to stimulus and cellular process. Ingenuity Pathway Analysis^TM^ (IPA) suggested that curcumin may exert its anticancer effects over multiple critical biological pathways including the EIF2, eIF4/p70S6K, mTOR signaling and mitochondrial dysfunction pathways. Functional validations confirmed that curcumin downregulates cellular protein synthesis, and induces autophagy, lysosomal activation and increased ROS production, thus leading to cell death.

The study of naturally-derived compounds holds great potential for therapeutic and medicinal applications. One example is that of the polyphenolic compound curcumin, an active component of turmeric (*Curcuma longa)* which has long been a staple of Asian cuisines as well as a traditional remedy to a wide range of illnesses^1^. It is now understood that curcumin exhibits highly pleiotropic effects including anti-cancer, anti-inflammatory and anti-infective properties^2^. Given its dietary role, it is also well-tolerated and safe to humans at dosages of up to 12 grams per day^1^,^3^. These characteristics have led to recent attention on the potential of curcumin as a novel anti-cancer therapy, with over 20 ongoing clinical trials for various cancers^1^,^4^. The pleiotropic nature of curcumin can be attributed to its ability to interact with a large number of cellular targets involved in multiple pathways^2^. It is therefore of great interest to identify the binding targets of curcumin in cancer cells. While reports have been made regarding potential targets of curcumin, the approaches have generally been restricted in scope or otherwise limited in terms of their applicability to living cells^5^. Recently, clickable small molecule probes have been used in identifying the targets of many bio-active natural products or drugs^6^,^7^,^8^,^9^,^10^,^11^,^12^,^13^. In this study, we comprehensively identified *in situ* the specific and direct protein binding targets of curcumin in a colon cancer cell line (HCT116), via the synthesis of a cell-permeable clickable curcumin probe (**Cur-P**) combined with quantitative chemical proteomics methods (Fig. 1a)^11^,^12^. Functional validations were subsequently carried out for key pathways identified as targets of curcumin, confirming the role of curcumin in downregulation of cellular protein synthesis, as well as the induction of autophagy, lysosomal activation and increased ROS production.

## Results

### Chemical synthesis of curcumin-based activity probe (Cur-P)

**Figure 1:**
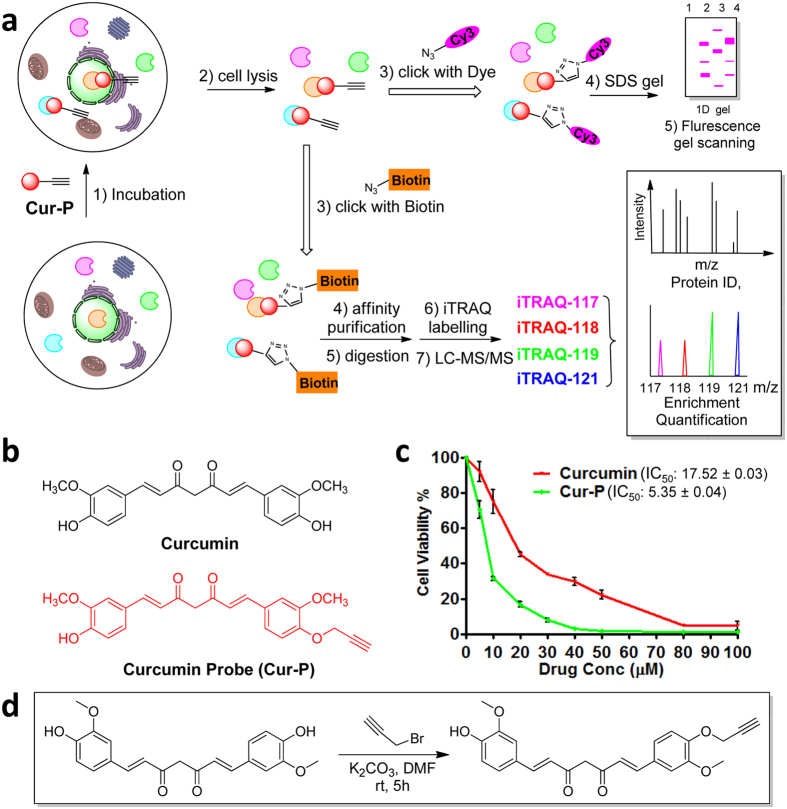
Overview of quantitative chemical proteomics approach for identifying the targets of curcumin. (**a**) Overall workflow for *in situ* profiling of potential curcumin targets in live cells. (**b**) The structure of curcumin and curcumin-based probe (**Cur-P**). (**c**) Dose-dependent inhibition of HCT116 cell proliferation by curcumin and **Cur-P**. (**d**) Synthetic scheme of Cur-P. Error bars represent s.d. in three independent replicates in (**c**).

Ideally, the design of the curcumin based probe must retain the active moiety of this natural product. Previous studies showed that the α, β-unsaturated ketone group of curcumin is critical for its biological activity^3^,^4^. Structure-activity relationship studies suggested that the derivative of curcumin at the hydroxyl group did not affect the active moiety of the drug^1^,^14^. To profile and identify the direct targets of curcumin, we introduced an alkyne group at the hydroxyl group position of curcumin to synthesize an activity-based probe, which enables the subsequent linking with a fluorescent dye or biotin (Fig. 1b,d, for synthesis, see supplementary information). Briefly, the curcumin probe (**Cur-P**) was readily synthesized by mono-alkylation of curcumin by propargyl bromide and its structure was verified by H-NMR, C-NMR and high resolution mass spectrometry. We have confirmed that the probe still retains the anti-cancer activities and **Cur-P** is even more potent than curcumin in HCT 116 colon cancer cells using the crystal violet assay (Fig. 1c). This is consistent with previous study which showed that the alkylation of ortho-phenolic OH group in curcumin and its derivatives can increase its anti-cancer activity^14^. However, all the OH modified derivatives share the same pharmacophore (α, β-unsaturated ketone group) which imply they have same mode of action^15^,^16^. To further confirm that **Cur-P** possesses similar biological activity as the parent compound, , the cells were treated with curcumin or **Cur-P** in parallel (*vide infra*) in the aftermentioned functional assays (ROS, Lysotracker staining and autophagy flux assay). No significant differences were observed between the drug and the probe (Supplementary Fig. S1).

### Fluorescence labelling profiling of curcumin binding proteins

**Figure 2:**
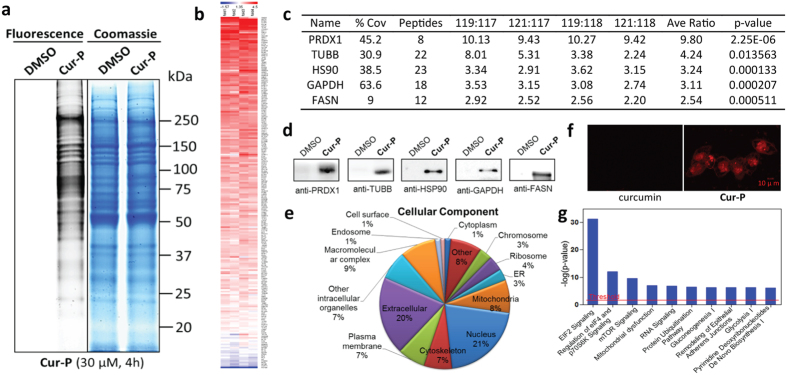
Quantitative chemical proteomics reveals curcumin-specific target proteins and involved pathways. (**a**) Left, fluorescence images of SDS-PAGE gel lanes of **Cur-P** (30 μM) – labeled HCT116 cells as well as the control cells treated with DMSO; Right, the same lanes stained with coomassie blue. (**b**) Heat map of the enrichment ratios of potential curcumin targets fulfilled the statistical requirement. An enlarged figure is shown in Supplementary Fig. S4 (**c**) The representative proteins identified by **Cur-P** in HCT116 cells (Sorted by average enrichment ratios). (**d**) Western-blotting validation of the selected **Cur-P** targets. (**e**) GO analysis of cellular localization of the **Cur-P** targets. (**f**) Cellular imaging of HCT116 cells incubated with 2 μM **Cur-P**. Cells were treated with Cur-P (2 μM) for 12 h, and then fixed and permeabilized for the click chemistry reaction. The fluorescence images were acquired with confocal microscopy. Scale bars, 10 μm. (**g**) Top canonical pathways that the curcumin protein targets are significantly over-represented.

To visualize the native cellular protein targets of curcumin, the labeling concentration of **Cur-P** was firstly optimized. Live HCT 116 cells were treated with increasing concentrations of **Cur-P** for 4 h before lysis. The lysate was then reacted with Rhodamine B-azide through click chemistry followed by SDS-PAGE and fluorescence scanning. The results showed a dose-dependent increase in fluorescence labeling and a satisfactory level of **Cur-P** labeling can be achieved with 30 μM of **Cur-P** (Supplementary Fig. S2), which was chosen for subsequent *in situ* gel-based fluorescence labeling (Fig. 2a) and pull-down experiments. To examine whether the **Cur-P** binds to similar protein targets in HCT 116 cell lines, we have also pre-treated the cell lysate with curcumin and then treated it with **Cur-P**. Curcumin pre-treatment essentially attenuated the probe labeling fluorescence signals, suggesting that **Cur-P** largely targets the same proteins as curcumin (Supplementary Fig. S3).

### Identification of curcumin targets by quantitative chemical proteomics

Next, iTRAQ-based quantitative chemical proteomics was performed to identify the targets of curcumin using **Cur-P**. To cope with the biological and experimental variations, two curcumin probes- and two DMSO-treated samples were analyzed as biological replicates. HCT116 cells were incubated with 30 μM **Cur-P** or DMSO (negative control) for 4 h before lysis. The lysate was then reacted with the biotin azide tag, followed by affinity enrichment using avidin beads. The beads were thoroughly washed and on-beads trypsin digestion was conducted. The derived peptides were reacted with iTRAQ reagents (control samples were labeled with 117 or 118; **Cur-P** treated samples were labeled with 119 or 121). The labeled samples were then pooled together and analyzed by LC-MS/MS to identify and quantify the target proteins. For specific protein targets, the iTRAQ reporter ions 119 and 121 have significantly higher intensities than 117 and 118, whereas for the non-specific binding and endogenously biotinylated proteins, the reporter intensities are similar (Fig. 1a). In our study, a total of 370 proteins were successfully identified and quantified using iTRAQ-based quantification coupled with activity-based proteome profiling (ABPP) (Supplementary Table 1). iTRAQ ratios of each identified protein were subjected to statistical test and only proteins identified with at least two peptides and have *p-* values less than 0.05 were considered statistically reliable hits, which resulted in a list of 212 proteins (Supplementary Table 2). The distribution of the enrichment ratios of these proteins is presented as a colored heat map in Fig. 2b and Supplementary Fig. S4. To differentiate specific binding targets from non-specific ones, we then applied a highly stringent cut-off threshold (iTRAQ ratio ≥2.5) to minimize potential false-positive targets. Consequently, we have identified 197 proteins as the specific targets of curcumin (Supplementary Table 3).

To further validate the identified proteins as the direct binding targets of curcumin, five representative proteins with enrichment ratios from 2.5 to 10 in our list were subjected to pull-down experiments with **Cur-P**, followed by immunoblotting with their respective antibodies (Fig. 2c,d). Results unequivocally confirmed the direct interaction between **Cur-P** and the protein targets.

### Pathway analysis of targets of curcumin

Subsequently, we performed Gene Ontology (GO) analysis of the curcumin targets. It was shown that the targets are broadly distributed in different parts of the cell, and especially enriched in the nucleus, mitochondria and plasma membrane (Fig. 2e). To confirm this, **Cur-P**-treated cells were examined with confocal microscopy to visualize the cellular distribution of curcumin targets (Fig. 2f). HCT116 cells were treated with **Cur-P**, fixed by paraformaldehyde and permeabilized by Triton X-100, and then conjugated with Rhodamine B-azide by click chemistry before confocal fluorescence imaging. **Cur-P**-treated cells showed high levels of fluorescence in the cell membrane and nucleus, whereas no fluorescence signal was observed in the control cells treated with DMSO (Fig. 2f). Thus, our imaging results are consistent with the GO analysis of our curcumin targets.

GO analysis also showed that the curcumin targets are involved in various biological functions including metabolic process, regulation, response to stimulus and cellular process, etc. (Supplementary Fig. S5). Ingenuity Pathway Analysis^TM^ (IPA) suggested that curcumin may exert its anticancer effects over multiple critical biological pathways including the EIF2, eIF4/p70S6K, mTOR signaling and mitochondrial dysfunction pathways (Fig. 2g). Other IPA analysis results were shown in Supplementary Fig. S6.

### Inhibition of *de novo* protein synthesis by curcumin

**Figure 3:**
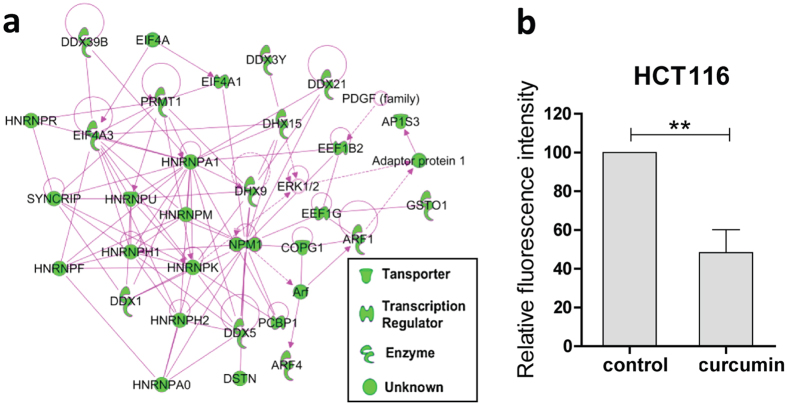
Curcumin inhibited *de novo* protein synthesis. (**a**) Network of curcumin target proteins (green nodes) affecting protein synthesis, revealed by Ingenuity Pathway Analysis^TM^ (IPA). (**b**) Curcumin treatment (5 μM, 16 h) significantly reduced the level of newly synthesized proteins in HCT116 cells. Data shown are mean ± SD from three independent experiments; **P < 0.01 (Student’s *t*-test).

Our pathway analysis highlighted elF2 signaling, regulation of elF4 and p70S6K signaling as well as mTOR signaling as the top three canonical pathways that the curcumin targets are involved in, suggesting that the cellular protein synthesis may be affected by curcumin (Figs 2g and 3a). Therefore, we first examined the changes of the protein synthesis levels of HCT116 cells upon curcumin treatment. We used azidohomoalanine (AHA), an artificial amino acid that can be incorporated into the newly synthesized proteins to monitor the level of protein synthesis^17^. The incorporated AHAs were then labeled with a fluorescence tag via click chemistry and the fluorescence intensity of the cells was measured by flow cytometry. As shown in Fig. 3b, curcumin (5 μM) treatment for 16 h caused a 50% reduction of the AHA signal intensity in HCT116 cells, indicating that curcumin can potently inhibit *de novo* protein synthesis.

### Induction of autophagy by curcumin

**Figure 4:**
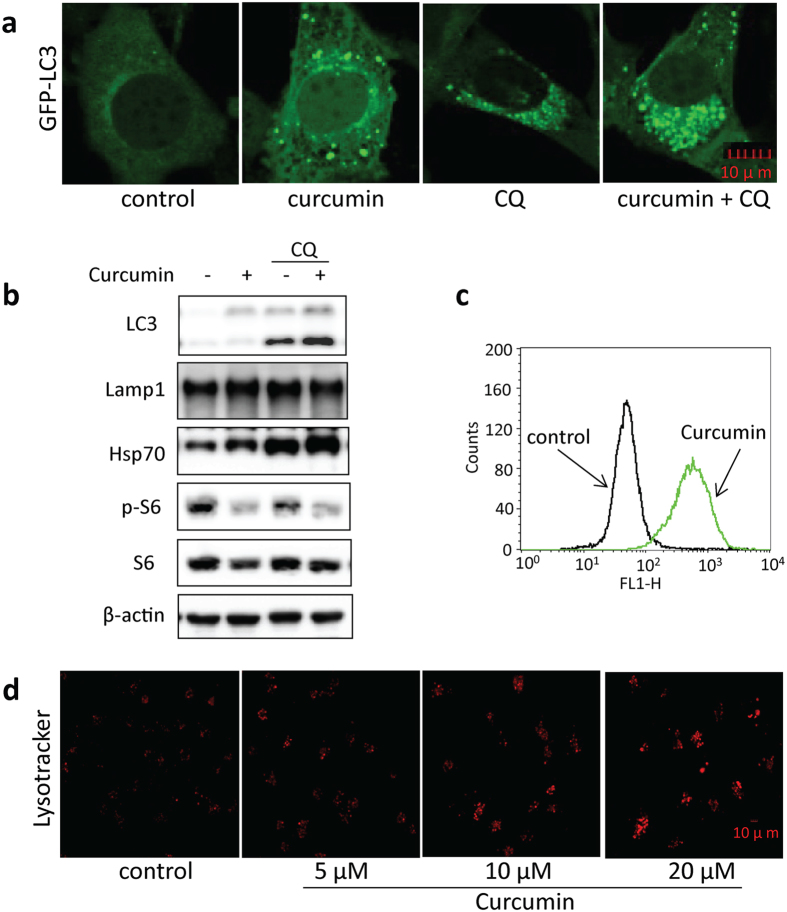
Curcumin induced autophagy and activated lysosomal function in cells. (**a**) Curcumin increased GFP-LC3 puncta in MEF cells. MEF cells stably expressing GFP-LC3 were treated with 20 μM curcumin for 12 h with or without 25 μM chloroquine. The GFP-LC3 distribution pattern was revealed with a confocal microscope. Scale bars, 10 μm. (**b**) HCT116 cells were treated with 20 μM curcumin with or without 25 μM chloroquine, followed by western-blot analyses of the cell lysates. β-actin was used as the loading control. (**c**) Curcumin increases ROS levels in HCT116 cells. Cells were treated with 20 μM curcumin for 12 h and then stained with DCFH-DA. The fluorescence signals were monitored with flow cytometry. (**d**) Dose-dependent increase of lysosomal acidification in HCT116 cells upon curcumin treatment. HCT116 cells were treated with curcumin (5, 10 or 20 μM) for 12 h followed by staining with LysoTracker Red DND-99 (50 nM) for 15 min. Scale bars, 10 μm.

As reflected in our pathway analysis, mTOR pathway was involved in curcumin-treated cells. mTOR (Mechanistic Target of Rapamycin) is a serine/threonine protein kinase that regulates multiple biological processes including cell growth, cell proliferation, cell motility, cell survival, and protein synthesis, etc^18^,^19^,^20^. Inhibition of protein synthesis by curcumin implies that mTOR signaling pathway may be inhibited. This is in agreement with previous studies showing that curcumin can inhibit phosphorylation of mTOR and its downstream effectors phosphorylation of p70 S6 kinase 1 (S6K1) and eukaryotic initiation factor 4E (eIF4E) binding protein 1 (4E-BP1), and disrupt the mTOR complex^21^,^22^,^23^,^24^. mTOR has been well known to negatively regulate autophagy via suppressing the ULK1 (the mammalian homolog of yeast Atg1) complex, which consists of ULK1, FIP200, ATG13 and ATG101^25^,^26^. Therefore, we hypothesized that curcumin treatment may enhance autophagy of the cells. To verify this hypothesis, the changes in mTOR activity and autophagy markers were examined by immunoblotting. As shown in Fig. 4a,b, curcumin treatment increased autophagy flux level in HCT116 and MEF cells, consistent with previous reports^27^,^28^,^29^. Western blot detection of increased autophagy marker LC3 level further confirmed curcumin as an autophagy inducer. In addition, the inhibitory effect of curcumin on mTOR activity was also verified by the suppression of S6 phosphorylation (Ser235/236), a downstream protein substrate of mTOR (Fig. 4b).

Our pathway analysis also highlighted the mitochondrial dysfunction which usually accompanied by the change of the ROS level. Given the role of reactive oxygen species (ROS) in autophagosome development and autolysosomal degradation, we further measured changes in ROS production following curcumin treatment. As indicated by Fig. 4c, curcumin increased ROS levels in HCT116 cells, consistent with previous reports by Lee *et al.*^30^ and suggesting that effects on ROS production could contribute to autophagic induction by curcumin.

At the late stage of autophagy, autophagosomes are fused with lysosome to form autolysosome for degradation. Under starvation condition or upon mTOR inhibition, lysosome is activated^31^. mTOR inhibition in curcumin-treated cells has been previously reported^21^,^22^,^23^,^24^, and confirmed by our pathway analysis and *de novo* protein synthesis inhibition assay. Here, we also determined changes in HCT116 cells at lysosomal level. As shown in Fig. 4d, LysoTracker staining showed a significant increase of cellular fluorescence intensity in HCT116 cells treated with different concentrations of curcumin, indicating enhanced acidification and lysosomal activation. In addition, we also showed that curcumin increased lysosomal membrane protein expression in HCT116 cells, such as Lamp1^32^ and Hsp70^33^ (Fig. 4b). Taken together, these results suggested that curcumin also acts by lysosomal activation to induce autophagy.

## Discussion

The mechanism of action (MOA) of curcumin has been widely studied. It has been reported to target multiple proteins such as transcription factors, kinases and cytokines, and affect multiple pathways/biological processes such as inhibition of protein synthesis, induction of autophagy and apoptosis, anti-inflammatory and antioxidant processes, etc.^1^,^4^,^34^,^35^. However, most of the prior reports focused on the study of individual pathway/process, thus would not provide a full picture of the MOA of curcumin. Comparative proteomics profiling approaches have been applied to examine the curcumin treated samples versus the controls and multiple proteins with abundance changes were identified. Although these studies provided a comprehensive overview of curcumin induced cellular changes, they could not distinguish primary alterations from the secondary effects^36^,^37^,^38^,^39^. Identification of direct binding targets of curcumin, on the other hand, provides clues on the initial and most important changes that are caused by the compound. Previously, curcumin has be tagged with biotin, or immobilized to a solid phase and then reacted with cell/tissue lysates^5^. The protein targets were then affinity purified, followed by gel-based separation and mass spectrometric identification. However, such *in vitro* studies may not truly reflect the actual drug-target interaction *in vivo*. Also, gel-based approaches were restricted by a limited dynamic range, thus only the most abundant protein targets were identified. Therefore, the results obtained were not comprehensive enough and no pathway and functional validation were conducted in those studies. In this context, we introduced a tiny alkyne group to curcumin to create an activity-based probe. Unlike the bulky biotin tag, the small alkyne group does not affect the compound’s ability to penetrate the plasma membrane thus the probe can be used to target the proteins *in vivo*. After cell lysis, a biotin tag was then conjugated to the protein targets using click chemistry, which facilitates affinity purification of the targets. LC-MS was used for the protein target identification, which has wider dynamic range compared to gel-based approaches, leading to a dramatic increase of the total number of curcumin targets identified. In addition, iTRAQ quantitative proteomics approach was applied to filter out the non-specific binding proteins and endogenously biotinylated proteins. This enhanced the reliability of the curcumin targets that we identified. In total, 197 proteins were confidently identified as curcumin binding targets in our study. Several pathways that the curcumin targets are involved in were validated, including protein synthesis inhibition, autophagy and ROS induction. These cellular changes upon curcumin treatment are consistent with previous literature, confirming the reliability of our curcumin target dataset. Our results thus provided a comprehensive overview into the MOA of curcumin for its anti-cancer therapeutic effects, which will be beneficial for its future clinical application. The stringent experimental conditions we set up in this study identified curcumin targets with high confidence, but excluded the possibility of identifying any potential non-covalent binding targets. To overcome this limitation, we are developing a new curcumin probe with a photo cross-linking group which can be used to capture the transient curcumin-protein interaction. It will also be interesting to determine the roles of individual covalent binding target in curcumin-mediated cancer cell killing in future. Our current approach can also be applied to study the curcumin targets in normal cell lines in parallel with the cancer cells and compared with quantitative proteomics, so that we can further fish out most critical targets for curcumin’s anti-cancer property. The studies can also be extended to animal models in future. Nevertheless, as our results showed that curcumin can covalently bind to hundreds of protein targets, further investigation would be required before seriously considering it as a potential drug candidate.

## Methods

### *In Situ* fluorescence labeling experiments

HCT116 cells were cultured in six-well plates until 80 – 90% confluence was reached. Then the cells were washed thrice with PBS. Cur-P (5–60 μM) in 2 ml of medium with a final DMSO concentration of 1% was added, and the cells were incubated at 37 °C with 5% CO_2_ for 4 h. Control treatment was done with the same volume of medium containing 1% DMSO. After treatment, the cells were washed with PBS and trypsinized to detach from the plate. The cells were pelleted, resuspended in PBS, washed, and subjected to sonication in 100 μl of PBS to lyse the cells. Centrifugation (10,000 rpm; 45 min) was applied to remove the insoluble fraction from the cell lysate. Bradford assay was used to determine the protein concentrations of the supernatants. Equal amounts (50 μg) of the extracted proteins were then subjected to fluorescence labeling. The click reaction was done by adding Rhodamine B -azide (10 μM), TCEP (1 mM), TBTA (100 μM), and CuSO4 (1 mM) to the lysate, followed by 2 h-incubation at room temperature. The labeled proteins were then acetone-precipitated and air-dried. The samples were then solubilized with 100 μl of 1× SDS loading buffer. Fifty microliter of sample was separated with 4–20% gradient SDS-PAGE gel. Typhoon 9410 laser scanner (GE Healthcare) was used to obtain the gel images, which were analyzed by Image Quant software.

### Curcumin target identification using quantitative chemical proteomics

In the subsequent target identification, two biological duplicates of Cur-P-treated samples and two control samples treated with DMSO were affinity-enriched and parallel digested and then labeled with iTRAQ reagents (119, 121, 117 and 118 respectively), followed by LC-MS/MS to identify and quantify the protein targets.

Briefly, the HCT116 cells were cultured in 150 mm culture dish until 80% confluence was reached. After removal of culture medium and washing twice with PBS, Cur-P (30 μM) in 20 ml of medium with a final DMSO concentration of 1% was added to the cells, followed by incubation for 4 h. Control treatments were performed with culture medium containing 1% DMSO. The media were discarded after treatment, and then the cells were subjected to PBS wash and trypsinization to detach from the plate. The cells were pelleted, washed with PBS, and sonicated to lyse in PBS. The insoluble fraction was removed by centrifugation (10,000 rpm; 45 min), and then the protein concentration of the supernatant was determined by Bradford assay. Equal amounts (4 mg) of the extracted proteins (two Cur-P-treated and two DMSO-treated samples) were conjugated with the biotin tags separately via click chemistry, by adding biotin-azide (10 μM), TCEP (1 mM), TBTA (100 μM) and CuSO4 (1 mM) followed by 4 h shaking. The reacted proteins were then acetone-precipitated and air-dried. The pellet was re-solubilized with 1 ml of PBS containing 0.1% SDS and then added 40 μl of Streptavidin beads, followed by 2 h-incubation at room temperature with gentle mixing.

### On-beads tryptic digestion

After washing three-time each with 1% SDS, 6M urea and PBS, 150 μL 20 mM Triethylammonium bicarbonate (TEAB) and 2 μL TCEP (100 mM stock solution) were added to re-suspend the beads, followed by incubation at 60 °C for 50 min. After addition of 1 μL MMTS (200 mM stock solution), the samples were kept at room temperature for 20 min. Trypsin (10 ng/μL) was then added and the samples were incubated at 37 °C for 16 h.

### iTRAQ labeling of the tryptic peptides

Labeling was performed using iTRAQ Reagent-8Plex reagent (SCIEX; Foster City, CA). The two negative controls and the two Cur-P pull-down samples were labeled with reagent 117, 118, 119 and 121 respectively. Briefly, after drying and reconstituting with 30 μL 0.5 M TEAB, the digested peptides were reacted with respective iTRAQ reagents for 3 h at room temperature. Afterwards, the labeled samples were pooled together and subjected to strong cation exchange and desalting. After desalting, the iTRAQ labeled peptide sample was dried and re-dissolved in 80 μl of 2% acetonitrile (ACN) containing 0.05% formic acid (FA).

### Nano LC−ESI-MS

For LC-MS/MS procedures, an Eksigent nanoLC Ultra and ChiPLC-nanoflex (Eksigent, Dublin, CA) system was used to separate iTRAQ labeled peptides with Trap-Elute configuration. Four microliter of the sample from the above section was injected into the system. The sizes of the trap and analytical columns are 200 μm × 0.5 mm and 75 μm × 150 mm respectively, and both were packed with ChromXP C18-CL, 3 μm (Eksigent, Germany). Mobile phase A was composed of 2% ACN and 0.1% FA, and mobile phase B was composed of 98% ACN and 0.1% FA. The flow rate was 300 nL/min. The following gradients were applied for the peptide separation: 5–12% B in 20 min followed by 12–30% B in 90 min, and then 30–90% B in 2 min. The columns were regenerated with 90% B for 5 min and 90–5% B for 3 min, and equilibrated with 5% B for 13 min^40^.

A TripleTOF 5600 system (SCIEX) was used to acquire the MS and MS/MS spectra. High resolution mode (>30000) was used to acquire the MS spectra at 400–1250 m/z range and the signals were accumulated for 250 ms for each spectrum. High sensitivity mode (resolution >15000) was used to acquire the MS/MS spectra. The “adjust CE when using iTRAQ Reagent” function was turned on. For each duty cycle, up to 20 precursors were chosen from the MS spectrum for MS/MS analysis/and the signals were accumulated for a minimum of 100 ms per spectrum. The dynamic exclusion time was set at 15 s.

### Protein identification and quantification

ProteinPilot™ 4.5 (SCIEX) was applied to protein identification and iTRAQ quantification, using SwissProt (2013_09, total 540958 sequences) as the database. The search parameter settings were: Cysteine alkylation with MMTS; Trypsin Digestion; TripleTOF 5600; Biological modifications. The identified proteins were grouped using the ProGroup algorithm to eliminate the redundancy. A decoy database search strategy was applied to determine the false discovery rate (FDR). Unused score >1.3 (corresponding to a protein confidence interval >95%) was applied as the cutoff threshold for protein identification, with a false discovery rate (FDR) of 0.33%.

## Additional Information

**How to cite this article**: Wang, J. *et al.* *In situ* Proteomic Profiling of Curcumin Targets in HCT116 Colon Cancer Cell Line. *Sci. Rep.* **6**, 22146; doi: 10.1038/srep22146 (2016).

## Supplementary Material

Supplementary Information

Supplementary Table 1

Supplementary Table 2

Supplementary Table 3
