# Supplementary material for: In situ Proteomic Profiling of Curcumin Targets in HCT116 Colon Cancer Cell Line
Source: Sci Rep. 2016 Feb 26;6:22146. doi: 10.1038/srep22146 (PMC4768257; doi:10.1038/srep22146)
Supplement: Supplementary Information [file srep22146-s1.doc]

Supporting Information

***In situ* Proteomic Profiling of Curcumin Targets in HCT116 Colon Cancer Cell Line**

Jigang Wang^1,2,3†*^, Jianbin Zhang^4†^, Chong-Jing Zhang^5^, Yin Kwan Wong^2^, Teck Kwang Lim^2^, Zi-Chun Hua^3^, Bin Liu^5^, Steven R. Tannenbaum^1,6*^, Han-Ming Shen^4*^, Qingsong Lin^2*^

^1^ Interdisciplinary Research Group in Infectious Diseases, Singapore-MIT Alliance for Research & Technology (SMART), Singapore, 138602

^2^ Department of Biological Sciences, National University of Singapore, Singapore 117543

^3^ The State Key Laboratory of Pharmaceutical Biotechnology, College of Life Science, Nanjing University, Nanjing, China, 210023,

^4^ Department of Physiology, Yong Loo Lin School of Medicine, National University of Singapore, Singapore, 117597

^5^ Department of Chemical and Biomolecular Engineering, National University of Singapore, Singapore, 117585

^6^ Departments of Biological Engineering & Chemistry, Massachusetts Institute of Technology, United States of America, 02139

[†] These authors contributed equally to this work.

* Correspondence and requests for materials should be addressed to:

Dr. Jigang Wang- Email: jigang@smart.mit.edu

Dr. Steven R. Tannenbaum- Email: srt@mit.edu

Dr. Han-Ming Shen- E-mail: han-ming_shen@nuhs.edu.sg

Dr. Qingsong Lin- Tel: 65-65167769. Fax: 65-67792486. E-mail: dbslinqs@nus.edu.sg

1.
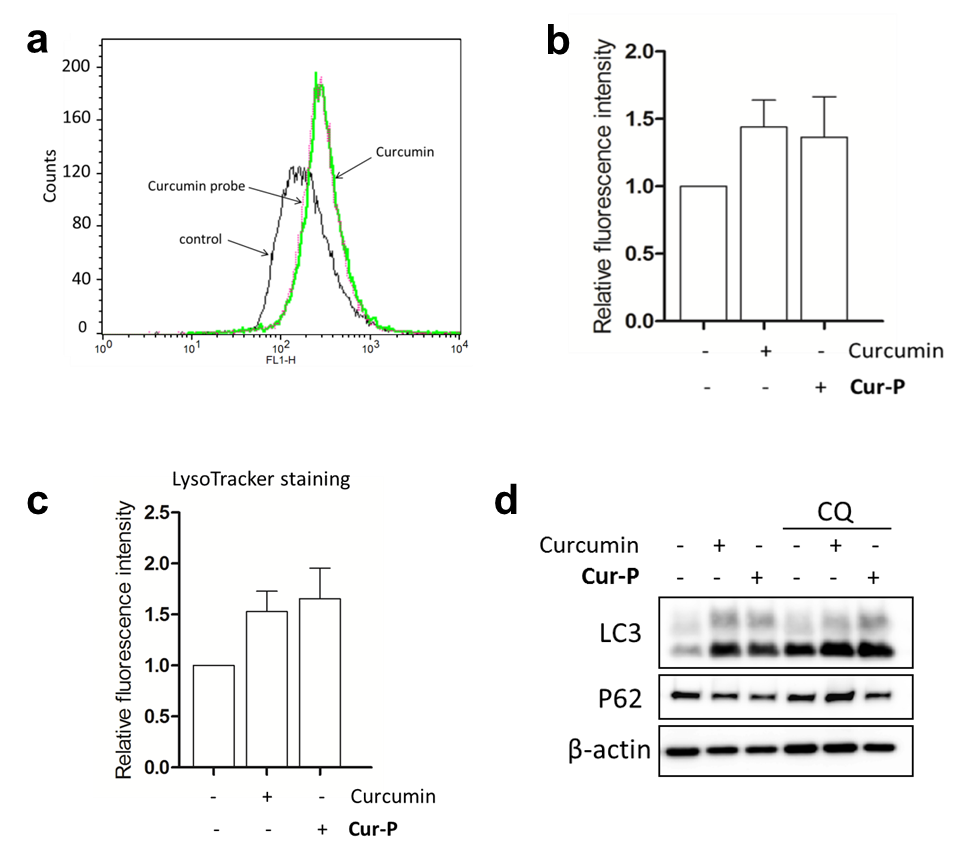
 **Supporting Figures**

**Supplementary Figure S1.** (a, b) HCT116 cells were treated with curcumin or **Cur-P** (10 μM) for 12 h and then stained with DCFH-DA. The redox state of cells was measured by flow cytometry. (c) HCT116 cells were treated with curcumin or **Cur-P** (10 μM) for 12 h and then stained with LysoTracker Red DND-99 (50 nM) for 15 min. The cellular fluorescence intensity was measured by flow cytometry. (d) HCT116 cells were treated with curcumin (20 μM) in the presence or absence of CQ (chloroquine, 25 μM) for 12 h and cell lysates were prepared for western blot. β-actin was used as the loading control.

**
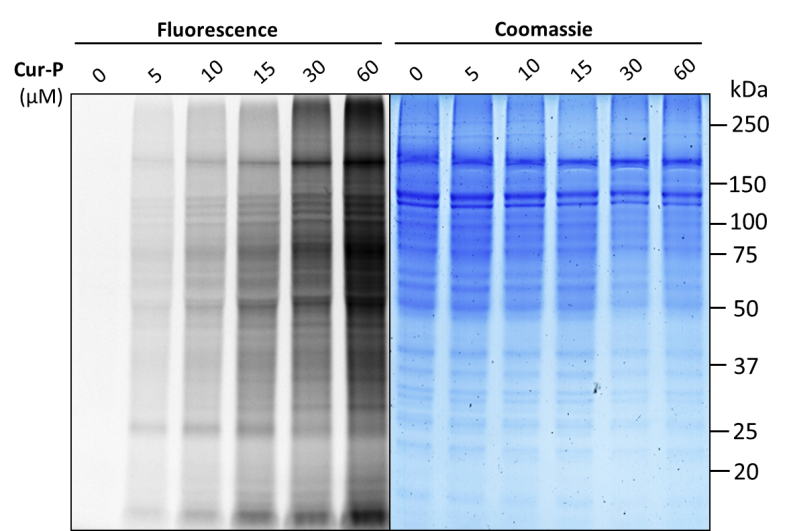
**

**Supplementary Figure S2.** *In situ* concentration dependent fluorescence labeling of curcumin binding targets by **Cur-P** (5 -60 µM, 4h).


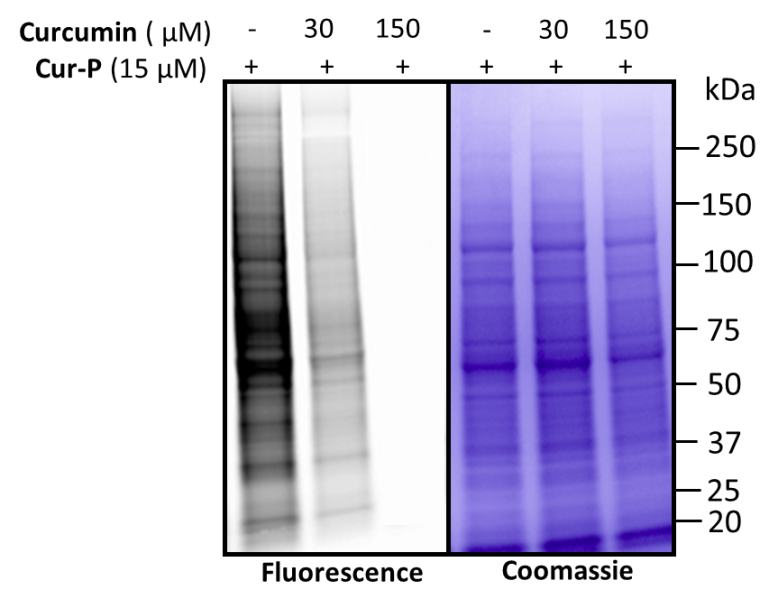


**Supplementary Figure S3.** The *in vitro* competition assay by in-gel fluorescence scanning. The HCT116 cell lysates were pretreated with curcumin (30 µM or 150 µM) for two h. Then the lysates were incubated together with **Cur-P** (15 µM) for another 4 h. A Rhodamine B -azide tag was conjugated to the probe labelled proteins by click chemistry. The protein lysates were then resolved by SDS-PAGE and visualized with fluorescence scanning.


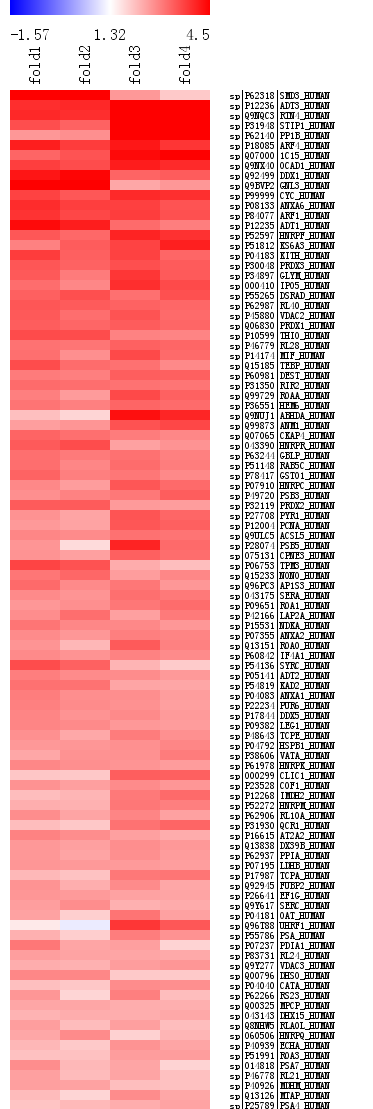

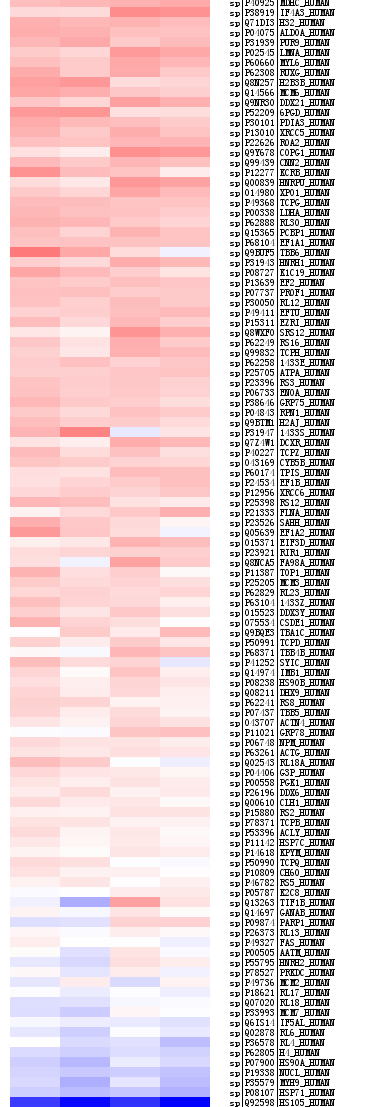


**Supplementary Figure S4.** Heat map of the enrichment ratio of potential curcumin targets fulfilled the statistical requirement. The most enriched proteins are displayed in red and least enriched proteins are in blue. The color scale for relative enrichment ratio is shown on top.

**
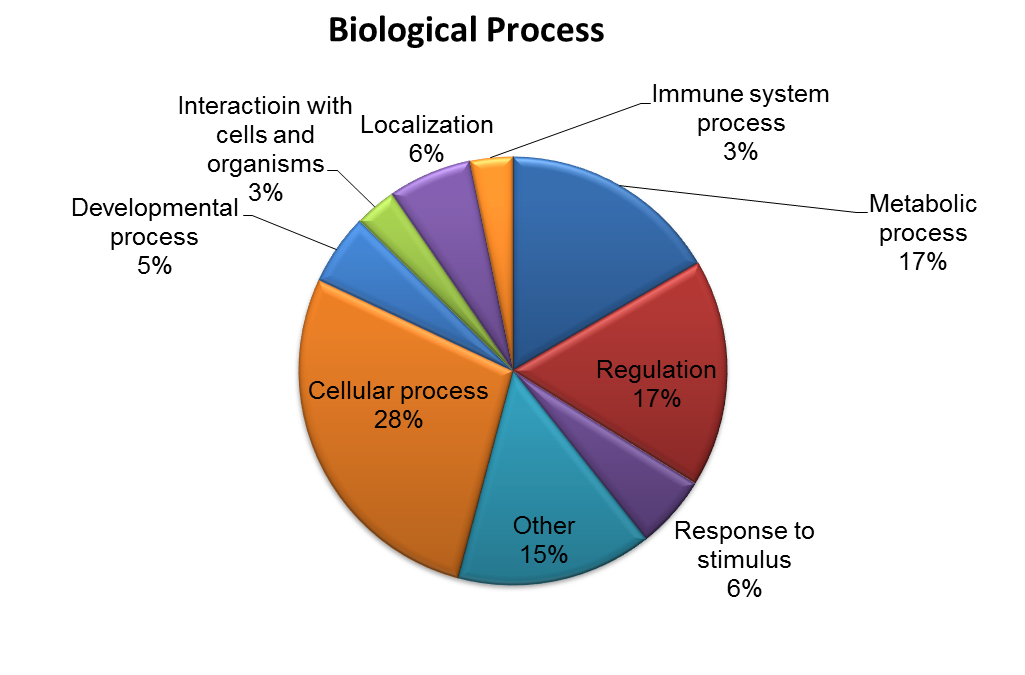
**

**Suplementary Figure S5.** Curcumin targets are involved in various biological processes.


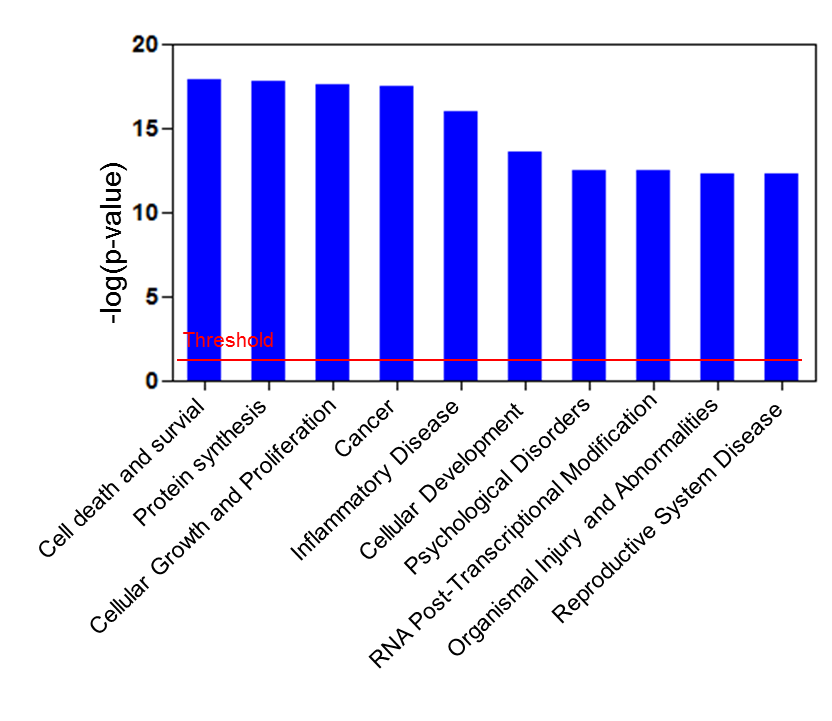


**Suplementary Figure S6.** Top molecular and cellular functional categories that the protein targets of curcumin are significantly over-represented.

**2. Supporting methods**

**General information**

Curcumin, Dimethyl sulfoxide (DMSO), acetonitrile (ACN), [streptavidin](http://www.google.com/search?hl=zh-CN&rls=com.microsoft:zh-cn:IE-SearchBox&rlz=1I7RNRN_en&spell=1&q=streptavidin&sa=X&ei=6XKsULSsBsbhrAfyloBg&ved=0CCEQvwUoAA) beads, methanol, urea, Trifluoroacetic acid (TFA), Tris (2-carboxyethyl) phosphine (TCEP) phosphoric acid, tris [(1-benzyl-1H-1,2,3-triazol-4-yl) methyl]amine (TBTA) and CuSO_4_ were obtained from Sigma-Aldrich (St. Louis, MO). Methyl methanethiosulfonate (MMTS) was purchased from Pierce (Rockford, IL). Dulbecco’s Modified Eagle Medium (DMEM containing 4500 mg/l D-glucose, without L-glutamine, sodium pyruvate, L-methionine, and L-cystine, Click-iT® AHA (L-azidohomoalanine) reagent, dialyzed [fetal bovine serum](http://products.invitrogen.com/ivgn/product/26400044?ICID=search-product), 4% formaldehyde in PBS, 0.5% TritonTMX-100 in PBS, 3% Bovine serum albumin (BSA) in PBS (pH 7.4), 1% SDS in 50 mM Tris-HCl (pH 8.0), and amino acid-free medium were purchased from Invitrogen (Carlsbad, CA). Trypsin (Sequencing grade) was obtained from Promega (Madison, WI). Rhodamine B-azide, Rhodamine B-alkyne and biotin-azide were obtained from Click Chemistry Tools, Scottsdale, AZ , USA. All the other reagents used were obtained from Sigma-Aldrich unless otherwise indicated. The antibodies used included: beta-tubulin (T9026), β-actin (A5441) and microtubule-associated protein 1 light chain 3 (LC3) (L8918) from Sigma Aldrich; PRDX1(#8732), HSP70 (#4872), HSP90 (#4874), FASN (#3189), LAMP1 (#9091), phospho-S6 (Ser235/236) (#4858), S6 (#2217), GAPDH (#5174) from Cell Signaling Technology (Danvers, MA).

## Synthesis of Cur-P

K_2_CO_3_ (414 mg, 3.0 mmol) and 3-bromoprop-1-yne (318 mg, 2.7 mmol) were added to a stirred solution of curcumin (1.0 g, 2.7 mmol) in DMF (20 mL), and continued to stir at room temperature for 5 h. The mixture was then diluted with H_2_O, extracted with EA, combined and concentrated. The residue was purified by pre-HPLC with PE:EA = 5:1 to give the title compound as a light yellow solid (300 mg, 27%). ^1^H NMR (400 MHz, CDCl_3_) δ 7.61 (d, *J* = 15.6 Hz, 2H), 7.08-7.15 (m, 3H), 6.93 (d, *J* = 8.4 Hz, 1H), 6.51 (d, *J* = 15.6 Hz, 2H), 6.49 (d, *J* = 15.6 Hz, 2H), 5.81 (s, 1H), 4.80 (d, *J* = 2.4 Hz, 2H), 3.94 (s, 3H), 3.94 (s, 3H), 2.54 (t, *J* = 2.4 Hz, 1H); ^13^C NMR (100 MHz, CDCl_3_) δ 183.6, 182.8, 149.8, 148.6, 147.9, 146.8, 140.6, 140.0, 129.3, 127.6, 122.8, 122.5, 121.9, 121.8, 114.8, 113.9, 110.5, 109.7, 101.2, 78.0, 76.1, 56.6, 55.9; LC-MS (APCI) calcd for [M+H]^+^: 407.1489, found: 407.1487.

**Scheme 1. Synthetic route of the curcumin probe (Cur-P)**

**Cell culture**

HCT116 cell line was from ATCC (Manassas, VA). The cells were maintained in modified McCoy's 5A medium with L-glutamine (Sigma, St. Louis, MO) supplemented with 10% fetal bovine serum ([Invitrogen](http://www.mcponline.org/cgi/redirect-inline?ad=Invitrogen), Carlsbad, CA) and 1× antibiotic/antimycotic ([Invitrogen](http://www.mcponline.org/cgi/redirect-inline?ad=Invitrogen)) at 37 °C with 5% (v/v) CO2.

**Inhibition of HCT 116 colon cancer cell proliferation**

To a 96-well plate, 20,000 cells were seeded into individual wells and allowed to attach for 24 h. The cells were then treated with curcumin or Cur-P at various concentrations for 24 h. After treatment, the media were discarded and the wells were rinsed with PBS. The cells were then incubated with 0.5% crystal violet in 20% methanol for 10 min. After the dye was removed, the wells were washed with PBS and allowed to dry. One percent SDS was added to the wells followed by incubation for 30 min to solubilize the dye, and the absorbance was measured at 550 nm.

**iTRAQ data analysis**

The identified proteins were first filtered by testing whether the mean log2 values of the four sets of Cur-P pull-down versus DMSO pull-down ratios were truly different from 0 (1-sample t-test). Only the proteins (n = 212) with p ≤ 0.05 with minimum two peptides were selected. Subsequently, the four sets of Cur-P pull-down *versus* DMSO pull-down ratios of these proteins were used to create a colored heat map (Fig. 2b) with MultiExperiment Viewer. Proteins with an enrichment ratio close to 1 are denoted in blue and are likely nonspecific-binding proteins. In contrast, proteins labeled in red showed enrichment ratios above 2.5, suggesting that they are likely the specific binding targets.

The stringent cutoff threshold applied (ratio ≥2.5) was to reduce the chance of selecting non-specific binding proteins for downstream analyses. Proteins identified based on a single peptide were considered less reliable and were removed from the list. Using these criteria, 197 proteins were selected as the potential targets of curcumin.

**Validation of drug target using Western Blot**

A Cur-P affinity pull-down sample was separated via 1D SDS-PAGE together with the DMSO pull-down sample. After SDS-PAGE, the proteins were transferred onto PVDF membranes (Bio-Rad). The blots were blocked with 5% (w/v) BSA in PBS with 0.1% Tween 20 (PBS-T) for 4 h at room temperature. The membranes were incubated with the following antibodies: anti-HSP90 (1:1000), anti-PRDX1 (1:1000), anti-FASN (1:1000), anti-β-tubulin (1:5000) as well as mouse anti-β-actin (1:4000). HRP-conjugated anti-rabbit (Pierce) or HRP-conjugated anti-mouse IgG (1:5000) (GE Healthcare) was used as a secondary antibody, and samples were incubated at room temperature for 3 h. The membrane was washed with PBS-T trice between each antibody incubation step, and ECL substrate (Pierce) was used for subsequent visualization.

**Pathway analysis of curcumin targets**

Ingenuity Pathway Analysis software (Ingenuity® Systems, Redwood City, CA) was applied to analyze the specific curcumin targets. Ingenuity Pathway Analysis core analysis was performed to obtain the canonical pathways and molecular networks that the curcumin targets were significantly over-represented.

Metabolic labeling of newly synthesized proteins with azidohomoalanine (AHA)

HCT116 cells were cultured in 6-well plates until 70~80% confluency was reached. After washing with PBS, the cells were continued to culture in L-methionine-free DMEM for 0.5 h so that the intracellular methionine reserves could be deleted. Afterwards, the cells were cultured in 10% FBS DMEM (methionine-free) containing AHA for designated time. The cells were then harvested and fixed with 4% formaldehyde (10 min) and permeabilized with 0.25% TritonTM X-100 (15 min) in PBS at room temperature. Following, the cells were added with Rhodamine B alkyne (10 µM), TCEP (1 mM), TBTA ligand (100 mM) and CuSO4 (1 mM, and incubated for 2 h at room temperature. Subsequently, the cells were washed with PBS containing 3% BSA after the reaction cocktail was removed. Flow cytometry was used to assess the nascent protein synthesis, and AHA fluorescence signal intensity was determined in the FL3 channel. The ratio of the fluorescence intensity of curcumin (5 μM, 16 h) treated cells to that of the control (DMSO treated) cells were calculated.

**Confocal microscopy**

Coverglass slide chambers (Lab-Tek, NUNC, 155411) were used to cultivate GFP-LC3-expressing stable MEFs for curcumin treatments. A confocal microscope (Olympus Fluoview FV1000, Olympus America Inc., PA) was used to examine the treated cells. Images were taken for the representative cells.

**Western blotting for autophagy markers**

After lysing the cells in sample lysis buffer (62.5 mM Tris, pH 6.8, 2% SDS, 25% glycerol), an equal amount of proteins was resolved by SDS-PAGE and transferred onto PVDF membrane. The membrane was then blocked with 5% non-fat milk, probed with designated primary and secondary antibodies, developed with the enhanced chemiluminescence method and visualized with ImageQuant LAS 500 (GE Healthcare).

**Estimation of intralysosomal pH**

LysoTracker was used to estimate the intralysosomal pH, following the manufacturer’s instruction. A confocal microscope (Olympus Fluoview FV1000) was used to monitor the fluorescence intensity and representative cells were selected and photographed.

**Detection of ROS level**

HCT116 cells were treated with curcumin (20 μM) for 12 h and then stained with DCFH-DA. Flow cytometry was applied to quantify the fluorescence signals.

**Cellular imaging of curcumin-binding proteins**

NuncTM Lab-TekTM coverglass slide chambers (Thermo Fisher Scientific; Waltham, MA) were used to cultivate the HCT116 cells. After 12 h Cur-P (2 μM) labeling, cells were washed with PBS, then fixed with 4% paraformaldehyde (10 min) and permeabilized with 0.25% TritonTM X-100 (20 min) in PBS at room temperature. After washing with PBS, and the cells were blocked with PBS containing 1% BSA for 25 min, and conjugated with Rhodamine B azide via click chemistry. A confocal microscope (Olympus Fluoview FV1000) was used to examine the cells and representative cells were selected and photographed.


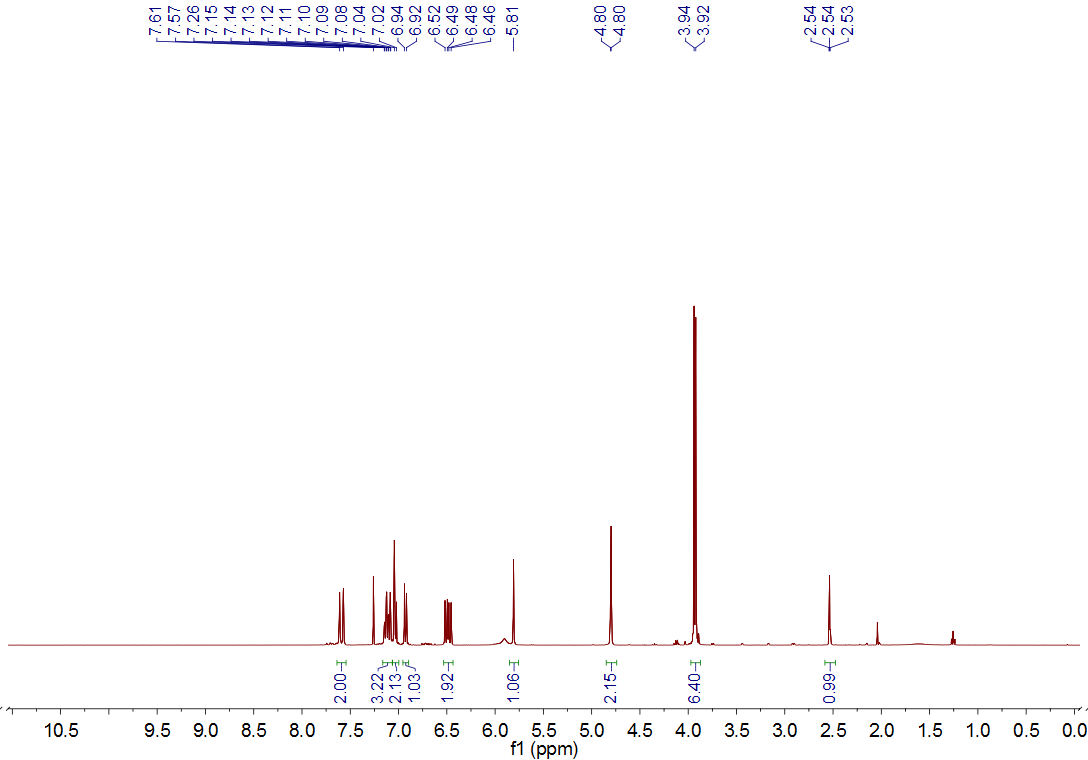


**^1^H-NMR of Cur-P in CDCl3**


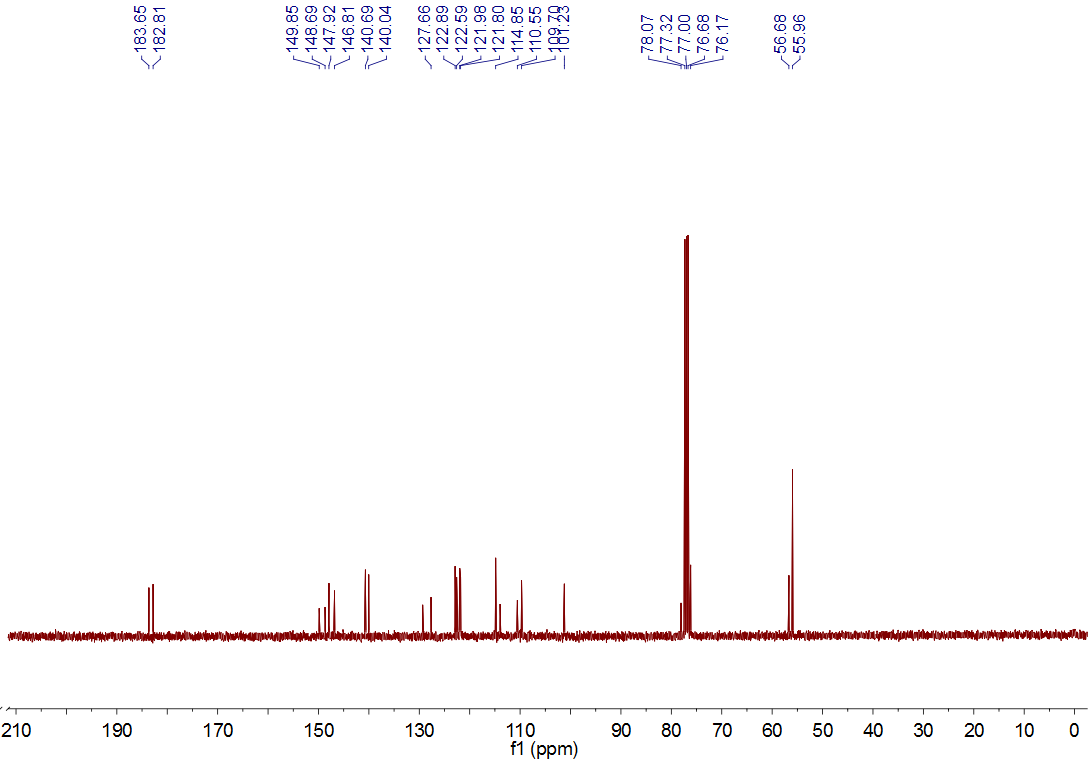


**^13^C-NMR of Cur-P in CDCl3**


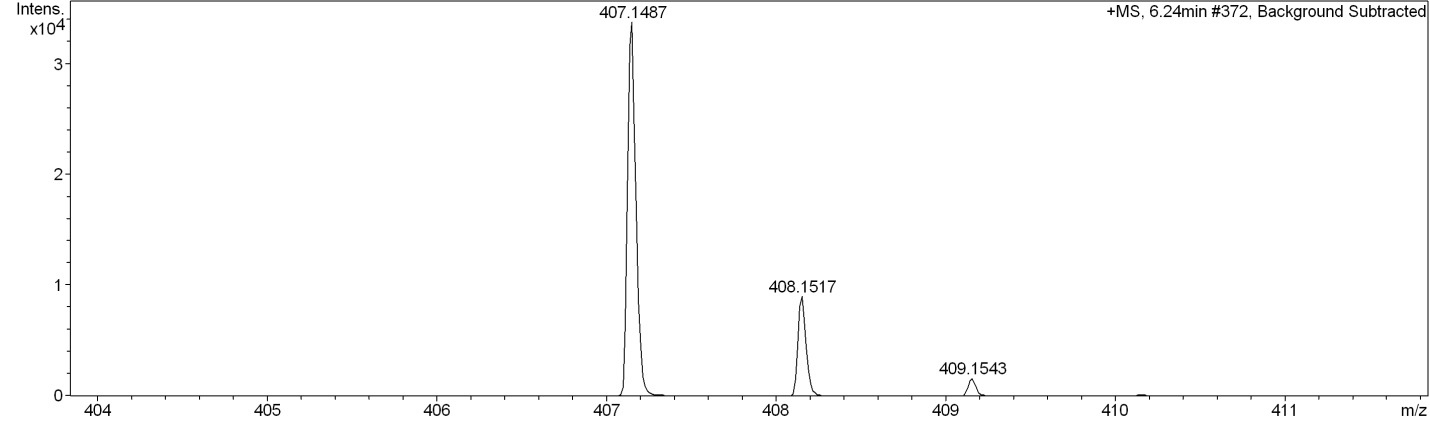


**High resolution mass spectrum of Cur-P**
